# Graded Defects in Cytotoxicity Determine Severity of Hemophagocytic Lymphohistiocytosis in Humans and Mice

**DOI:** 10.3389/fimmu.2013.00448

**Published:** 2013-12-16

**Authors:** Birthe Jessen, Tamara Kögl, Fernando E. Sepulveda, Genevieve de Saint Basile, Peter Aichele, Stephan Ehl

**Affiliations:** ^1^Centre of Chronic Immunodeficiency, University Medical Center Freiburg, University of Freiburg, Freiburg, Germany; ^2^Department for Medical Microbiology and Hygiene, Institute of Immunology, University Medical Center Freiburg, University of Freiburg, Freiburg, Germany; ^3^U768, INSERM, Paris, France; ^4^Institut Imagine, Université Paris Descartes-Sorbonne Paris Cité, Paris, France; ^5^Centre d’Etudes des Déficits Immunitaires, Assistance Publique-Hôpitaux de Paris, Hôpital Necker, Paris, France

**Keywords:** cytotoxicity, hemophagocytic lymphohistiocytosis, inflammation, CTL, virus persistence, antigen persistence

## Abstract

Primary hemophagocytic lymphohistiocytosis (HLH) is a life-threatening disease of hyperinflammation resulting from immune dysregulation due to inherited defects in the cytolytic machinery of natural killer and T cells. In humans, mutations in seven genes encoding proteins involved in cytolytic effector functions have so far been identified that predispose to HLH. However, although most affected patients develop HLH eventually, disease onset and severity are highly variable. Due to the genetic heterogeneity and variable time and nature of disease triggers, the immunological basis of these variations in HLH progression is incompletely understood. Several murine models of primary HLH have been established allowing to study HLH pathogenesis under more defined conditions. Here we directly compare the clinical HLH phenotype in six HLH-prone mouse strains with defects in the granule-dependent cytotoxic pathway. A severity gradient of HLH manifestations could be identified that is defined by the genetically determined residual lytic activity of cytotoxic T lymphocytes (CTL) and their ability to control lymphocytic choriomeningitis virus, which was used as a trigger for disease induction. Importantly, analysis of cohorts of HLH patients with severe bi-allelic mutations in the corresponding genes yielded a similar severity gradient in human HLH as reflected by the age at disease onset. Our findings define HLH as a threshold disease determined by subtle differences in the residual lytic activity of CTL.

## Introduction

Primary hemophagocytic lymphohistiocytosis (HLH) is a rare life-threatening syndrome of hyperinflammation due to genetic defects in the perforin-dependent granule exocytosis pathway of natural killer (NK) and T cells (1–4). The HLH syndrome is characterized by uncontrolled inflammatory and immunopathological processes in various tissues as a result of infiltrating, excessively activated T cells, NK cells, and macrophages, accompanied by a massive cytokine production (IFN-γ, TNF, IL-6, IL-18) (5–7). Due to this loss in immune homeostasis, HLH patients present with prolonged fever, hepatosplenomegaly, severe cytopenia, and frequently with neurologic manifestations. In addition, elevated serum levels of ferritin, triglycerides, soluble CD25 (IL-2 receptor α chain), and liver enzymes, as well as hemophagocytosis in various tissues and reduced cytolytic activity of lymphocytes are characteristic criteria for HLH (Table 1) (8). Typically, patients with primary HLH develop disease in early childhood with a poor prognosis in the absence of therapeutic intervention (9–11).

**Table 1:** **Diagnostic criteria for HLH**.

Fever
Cytopenia in at least two cell lineages
Hyperferritinemia
High sCD25 (sIL-2Rα) concentration
Hypertriglyceridemia and/or hypofibrinogenemia
Splenomegaly
Hemophagocytosis
Low/absent natural killer (NK) cell cytotoxicity

Traditionally, “familial HLH” (FHL) has been defined as a genetic disease, in which the predisposition to HLH is the dominant feature (*PERFORIN* deficiency, *MUNC13-4* deficiency, *SYNTAXIN-11* deficiency, and *MUNC18-2* deficiency) (12–17), while “immunodeficiencies with albinism” (Chediak–Higashi syndrome (CHS) or *LYST* deficiency, Griscelli syndrome type 2 (GS2) or *RAB27A* deficiency, and Hermansky–Pudlak syndrome type 2 (HPS2) or *AP3b1* deficiency) (18–22) combine this predisposition with clinical manifestations of albinism and variable degrees of other immune cell and platelet dysfunction (23–28). From a pathophysiological viewpoint, this distinction is arbitrary. First, all genes mutated in these two groups of conditions are critically involved in the biogenesis, intracellular transport, release, and function of perforin-containing lytic granules of NK and T cells (1). Second, it becomes increasingly obvious that defects in platelets and other immune cells such as neutrophils or mast cells are also observed in diseases currently classified as FHL (29–33). Because the genetic predisposition to HLH is the dominant life-threatening clinical feature in all of these diseases, we prefer to classify them collectively as familial HLH syndromes (FHL syndromes).

While the overall pattern of clinical manifestations of HLH in patients with the different FHL syndromes is quite characteristic, onset of disease, severity of clinical symptoms, and duration of disease-free remission periods are highly variable (31, 34–36). This depends not only on the affected gene, but also on the nature of the mutation (null or hypomorphic) and the time point and nature of exposure to predominantly infectious triggers that can elicit HLH in predisposed individuals. In addition, in >60% of patients with FHL syndromes, no clear trigger for HLH can be identified and it is still a matter of debate whether an exogenous trigger is needed for disease induction at all (37–40). This variability makes it difficult to define the *a priori* risk of an individual patient to develop HLH in the different human FHL syndromes. A study of additional functional parameters may help to improve the predictability of HLH progression. For example, it is so far not clear, in what hierarchy the dysfunction of the different affected proteins becomes limiting for *in vivo* cytotoxicity.

In this context, animal models of FHL syndromes have proven useful to analyze the pathogenesis of HLH under more defined conditions. In 2004, Jordan et al. reported that following lymphocytic choriomeningitis virus (LCMV) infection as initial trigger, perforin-deficient (*PKO*) mice develop the full clinical picture of HLH as it is described for FHL2 patients (41). It was demonstrated that hyperactive cytotoxic T lymphocytes (CTL) and high levels of IFN-γ are the driving force behind the development of fatal HLH in *PKO* mice. Non-fatal HLH was observed after LCMV infection of *Jinx* mice (model for FHL3) (42), *STX-11*-deficient mice (model for FHL4) (43, 44), *ashen* mice (model for GS2) (45), *souris* mice (model for CHS) (46), and *pearl* mice (model for HPS2) (20). Although some of these strains were compared directly in these publications, the different mouse models were not analyzed in parallel under identical experimental conditions with standardized immunological and clinical criteria for HLH. Therefore, the relative risk for HLH development in these models in relation to the individual genetic defect and its consequences for cytotoxicity have not been fully defined. Moreover, the role of virus control and a potential contribution of the various proteins in processes other than cytotoxicity to the pathogenesis of HLH remain controversial.

In the present study we therefore performed a comprehensive comparative analysis of the clinical and immunological HLH phenotype in six different mouse models of FHL syndromes. In addition, recently published results on HLH severity (as determined by age at onset of HLH) in patients with FHL syndromes due to severe bi-allelic mutations were extended to additional genetic conditions (44). We discuss our results in the context of the overall value of LCMV-induced HLH in various murine cytotoxicity mutants for the understanding of human FHL syndromes and point out some key questions to be addressed in human and mouse models in the future.

## Results

### HLH severity differs in various mouse models of FHL syndromes

To analyze the impact of different defects in the cytotoxicity pathway of T and NK cells on HLH development, we assessed HLH parameters following intravenous LCMV infection in six established and previously described HLH-prone mouse models under identical experimental conditions with standardized read-out systems. The following mouse strains were used in this study: two mouse models for CHS carrying different mutations in the *Lyst* gene – *beige* and *souris* mice – (46), one mouse model for HPS2 deficient in AP-3 – *pearl* mice – (20), a *Rab27a*-deficient mouse model for GS2 – *ashen* mice – (45), one model for familial hemophagocytic lymphohistiocytosis (FHL) 2 deficient in perforin – *PKO* mice – (41, 47), and one model for FHL4 deficient in syntaxin-11 – *STX-11KO* mice – (43, 44). As previously described, none of these mutant mouse strains develop disease spontaneously. Infection with LCMV was used to induce disease.

**Figure 1:**
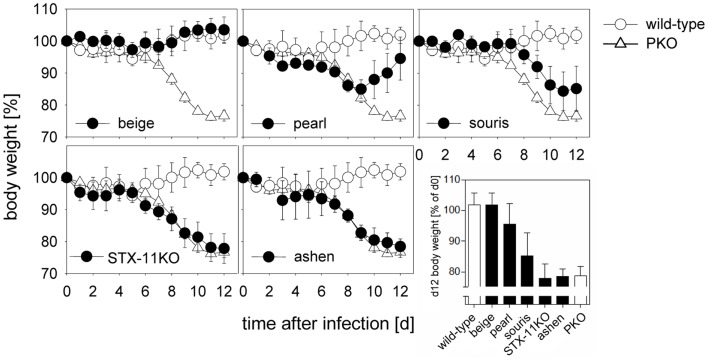
**Degree of weight loss after LCMV infection of different HLH-prone mouse models depends on the affected gene**. Mice were infected with 200 pfu LCMV i.v. Percent weight loss of initial body weight is depicted over the course of 12 days. Body weight of mutant mice (*beige, pearl, souris, STX-11KO, ashen*/filled circles) in comparison with *wild-type* (open circle) and *PKO* (open triangle) mice are shown. The graph depicted on the lower right illustrates a direct comparison of body weight loss of all mouse groups on day 12 after LCMV infection.

Following LCMV infection, mice were weighed and ear temperature was taken daily. Mice were analyzed either at day 8 or at day 12 after infection and all eight criteria (Table 1) defined by the HLH study group of the Histiocyte Society for the diagnostic evaluation of patients with suspected HLH were determined (8). A drop in ear temperature due to circulatory centralization was taken as an equivalent of fever in humans. In addition, we measured lactate dehydrogenase (LDH) and glutamate dehydrogenase (GLDH) reflecting liver damage and IFN-γ serum levels, which have been shown to correlate well with HLH activity in mice (41, 48). Weight loss as a rough, but easily accessible measure of disease revealed a clear hierarchy of HLH severity in the six mutant mouse strains (Figure 1). While there was no weight loss in *wild-type* and *beige* mice, weight loss was transient until day 8 in *pearl* mice, progressive but moderate in *souris* mice and equally severe in *STX-11KO, ashen*, and *PKO* mice.

A similar hierarchy of disease severity was observed, when the formal diagnostic HLH criteria were evaluated on day 12 (Table 2). LCMV infection of *wild-type* mice led to splenomegaly and rare hemophagocytosis, but the other HLH criteria were not fulfilled. *Beige* mice, carrying a hypomorphic mutation in the *lyst* gene, in addition had low NK cell cytotoxicity, but no other HLH features (Table 2). *Pearl* mice in addition had cytopenia, elevated ferritin, liver enzymes, and IFN-γ and thus fulfilled 5/8 diagnostic criteria at day 8 after infection (Table A1 in Appendix), but – as reported – disease was transient and the criteria were not fulfilled at day 12 (Table 2). All other strains fulfilled 7/8 (apart from elevated triglycerides) or 8/8 diagnostic criteria at day 12 and additionally had elevated liver enzymes and IFN-γ levels, which were more pronounced at day 8 after infection. Nevertheless, some notable differences were observed: first, the drop in temperature and alterations in hemoglobin, ferritin, sCD25, and triglycerides were less severe in *souris* mice than in the other three mouse strains – *STX-11KO, ashen, PKO* – although IFN-γ levels were similar or even higher. Second, alterations in platelet counts, ferritin, and sCD25 were less pronounced in *STX-11KO* than in *ashen* or *PKO* mice. Third, levels of ferritin and IFN-γ on day 8 after infection were higher in *PKO* than in *ashen* mice. Finally, previous experiments assessing survival beyond day 12 have shown that disease is usually lethal in *PKO* mice, sometimes lethal in *ashen* mice and not lethal in any of the other investigated mutant mouse strains (data not shown). Overall, HLH severity as assessed by weight loss, survival, and HLH criteria showed the following hierarchy: *wild-type* < *beige* (no HLH) < *pearl* (transient HLH) < *souris* < *STX-11KO* < *ashen* < *PKO* (full HLH).

**Table 2:** **Analysis of HLH diagnosis parameters in various HLH-prone mouse models on day 12 after LCMV infection**.

HLH parameter	Wild-type	Beige	Pearl	Souris	Syntaxin-11KO	Ashen	PKO
Fever/hypothermia	37.0 ± 0.4	37.0 ± 0.4	37.0 ± 0.4	35.4 ± 0.8*	34.7 ± 0.7*	34.1 ± 0.3*	34.4 ± 0.9*
WBC (×10^3^/μL)	10.3 ± 3.4	14.3 ± 7.0	13.7 ± 5.5	6.9 ± 3.5*	3.84 ± 2.1*	6.1 ± 3.5*	4.1 ± 2.0*
HGB (g/dL)	11.3 ± 1.5	10.6 ± 1.2	10.7 ± 1.2	6.5 ± 1.4***	3.8 ± 0.6***	3.0 ± 0.6***	4.6 ± 1.0***
PLT (×10^3^/μL)	839 ± 180	803 ± 213	441 ± 11***	253 ± 75***	507.1 ± 156.5***	221 ± 151***	228 ± 114***
Ferritin (ng/mL)	525.2 ± 199.4	508.6 ± 105.7	817.7 ± 296.3	1009.0 ± 158.2	1624.3 ± 761.9***	3202.0 ± 568.5**	2951.1 ± 2098.6***
sCD25 (pg/mL)	271.9 ± 121.6	291.1 ± 46.0	321.3 ± 195.3	631.7 ± 166.4***	709.1 ± 98.3***	833.3 ± 238.6***	823.5 ± 105.2***
Triglycerides (mg/dL)	71.1 ± 18.7	75.3 ± 26.2	88.7 ± 12.3	82.5 ± 35.4	216.0 ± 69.0***	146.7 ± 37.8	111.4 ± 62.0
Hemophagocytosis (liver)	(+)	**+**	**++**	**+++**	**+++**	**+++**	**+++**
Splenomegaly	**+**	**+**	**+**	**+**	**+**	**+**	**+**
NK cell cytotoxicity	Normal	***↓↓***	***↓***	***↓↓↓***	***↓↓***	***↓↓↓***	***↓↓↓***
Additional parameter
LDH (U/L)	502.7 ± 171.7	493.3 ± 161.2	1379.0 ± 1231.2	1464.7 ± 422.5*	978.0 ± 692.8	2053.3 ± 871.8*	1944.2 ± 1535.7**
GLDH (U/L)	11.1 ± 3.7	15.8 ± 6.8	121.9 ± 293.76*	181.6 ± 136.6***	113.5 ± 98.9***	345.9 ± 93.9***	156.1 ± 103.1***
IFN-γ (ng/mL)	b.d.	b.d.	b.d.	23.6 ± 6.2***	23.5 ± 12.9***	38.0 ± 12.5***	26.2 ± 16.7***
**HLH**	**NO**	**NO**	**Transient**	**YES**	**YES**	**YES**	**Lethal**

*Red fields indicate fulfilled HLH criteria*.

*The red font accentuates the final disease phenotype*.

***p < 0.001; **p < 0.01; *p < 0.05 (ANOVA; statistically significant differences compared to day 12 values of wild-type mice).

*b.d. below detection limit*.

### Disease development in HLH mouse models correlates with virus persistence

**Figure 2:**
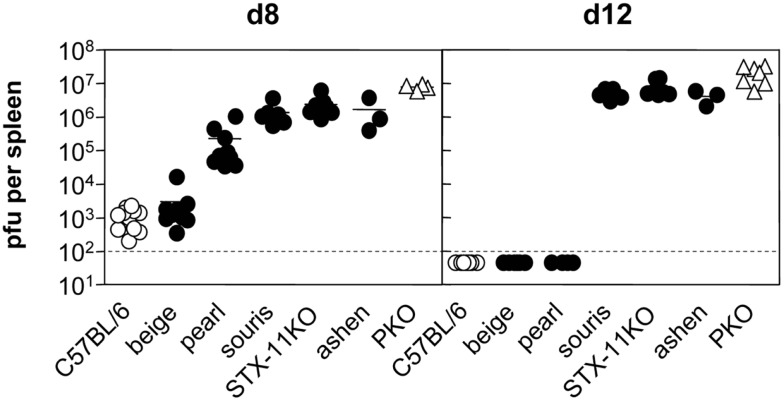
**Virus control following LCMV infection differs between various HLH-prone mouse models**. Mice were infected with 200 pfu LCMV i.v. On day 8 and day 12 after LCMV infection, viral titers in spleen of *beige, pearl, souris, STX-11KO*, and *ashen* mice (filled circle) in comparison to *wild-type* (open circle) and *PKO* (open triangle) mice were determined.

A comparison of virus titers in the spleen of the different HLH-prone mouse strains confirmed that virus persistence is one of the key characteristics of HLH development. Mice that showed no signs of disease such as *wild-type* and *beige* mice were able to reduce virus titers until day 8 and no virus was detectable at day 12 (Figure 2). In *pearl* mice, the transient HLH at day 8 was associated with a delay in virus control at this time point. When these mice recovered eventually, virus elimination was achieved. All of the mice fulfilling the criteria of HLH – irrespective of disease severity – failed to reduce or eliminate the virus until day 12 (Figure 2) and were persistently infected with similar titers in the spleen. Thus, virus persistence appears to be a prerequisite for the development of HLH in the various mouse models of impaired cytotoxicity, but disease severity does not correlate with titers of persisting virus.

### Graded defects in CTL cytotoxicity determine outcome of disease

**Figure 3:**
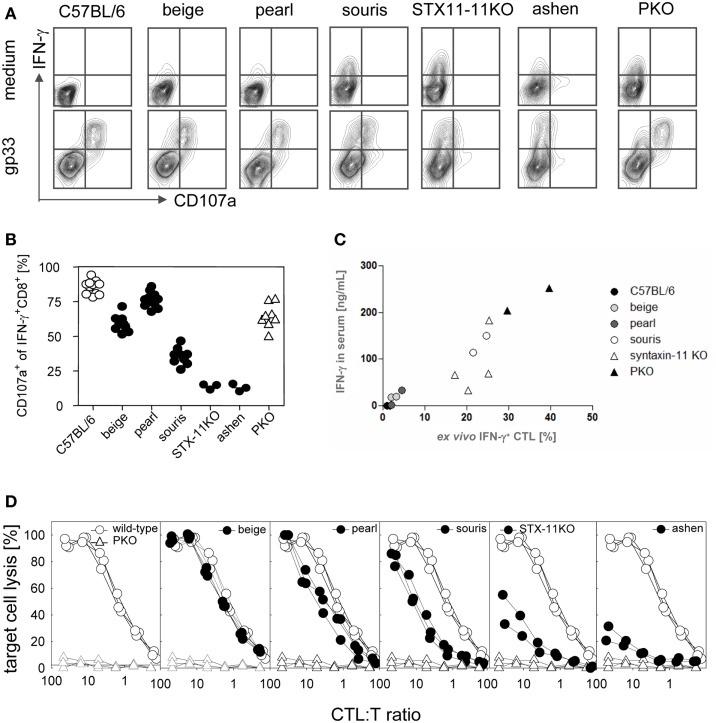
**Cytotoxic T lymphocytes degranulation and cytotoxicity are differently impaired in various HLH-prone mouse models**. Mice were infected with 200 pfu LCMV i.v. On day 8 after LCMV infection, spleen cells of mutant mice (*beige, pearl, souris, STX-11KO, ashen*/filled circles) and *wild-type* (open circle) and *PKO* (open triangle) mice were restimulated with gp33 peptide or were left in medium without peptide. Surface expression of CD107a of IFN-γ^+^CD8^+^ T cells was determined by flow cytometry as illustrated by **(A)** representative FACS plots. **(B)** Percentage of CD107a expressing IFN-γ^+^CD8^+^ T cells after restimulation with gp33 peptide. **(C)** Correlation of percentage of CTL expressing IFN-γ without restimulation *ex vivo* with the IFN-γ levels in sera of the various HLH-prone mutant mice. **(D)** CTL cytotoxicity was determined in a ^51^Cr-release assay by using LCMV-infected MC57 target cells.

As extensively discussed before (47, 49–53), the cytotoxicity of CD8 T cells is the major factor in virus control following LCMV infection and the major determinant in preventing HLH development (41, 43, 45). Here, we directly compared CTL degranulation and CTL cytotoxicity in the six different HLH-prone mouse strains on day 8 after LCMV infection. First, we analyzed the degranulation capacity of virus-specific CTL in the different mouse strains by quantifying the expression of CD107a on IFN-γ positive CTL upon antigen-specific *in vitro* stimulation with gp33 peptide, the immunodominant epitope of LCMV. CTL from the four mutant mouse strains that developed the full picture of HLH – *souris, STX-11KO, ashen, PKO* – continuously produced high levels of IFN-γ even in the absence of stimulation (Figure 3A, upper panel), while this was not the case in the other strains. Interestingly, the grade of *ex vivo* IFN-γ expression of CTL correlated very well with IFN-γ levels in serum and disease severity (Figure 3C). In contrast, the grade of the degranulation defect did not completely reflect the observations on disease severity. The degranulation defect was more pronounced in *beige* (no HLH) than in *pearl* mice (transient HLH) and no difference could be found in the degranulation defect between *STX-11KO* and *ashen* mice. Unexpectedly, a mild reduction in degranulation was also observed in *PKO* mice (Figure 3B), although a role for perforin in the process of granule exocytosis has so far not been described. Analyzing the *ex vivo* cytolytic activities of CTL from the different mouse strains revealed a graded impairment of cytotoxicity from beige to *PKO* CTL that perfectly reflected distinct disease severity (Figure 3D). In addition, as reported previously, the cytotoxicity defect was more pronounced in *PKO* than in *ashen* or *STX-11KO* mice.

**Figure 4:**
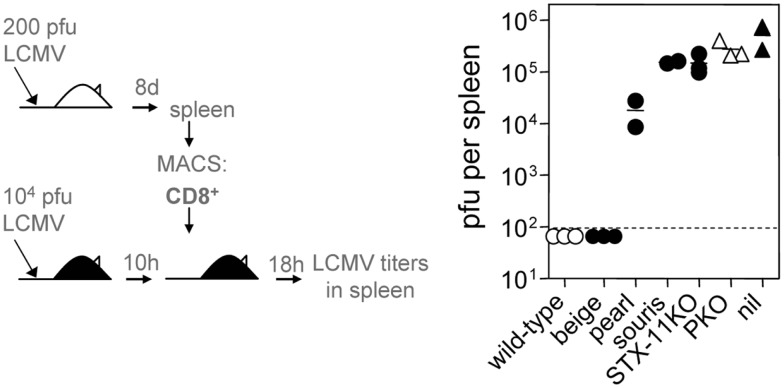
**Subtle differences in CTL cytotoxicity of various HLH-prone mouse models determine virus control**. On day 8 after 200 pfu of LCMV infection, splenic CD8^+^ T cells of mutant mice (*beige, pearl, souris, STX-11KO, ashen*/filled circles), *wild-type* (open circle), and *PKO* (open triangle) mice were MACS purified and 2 × 10^6^ of MACS purified CD8^+^ T cells were transferred into *wild-type* C57BL/6 mice that had been infected with 10^4^ pfu LCMV 10 h before. After 18 h of CD8^+^ T cell transfer, viral titers in spleen were determined (nil: infected *wild-type* C57BL/6 without cell transfer).

In a next step, we functionally evaluated the cytotoxic activity of CTL derived from the different HLH-prone mouse strains in an *in vivo* assay of virus control (50). For this, CTL were adoptively transfered into *wild-type* mice that had been infected with LCMV 10 h previously (Figure 4). In this assay, CTL from *wild-type* mice eliminated the virus from the spleen within 18 h, while CTL from the four strains that developed the full picture of HLH – *souris, STX-11KO, ashen, PKO* – had no impact on viral titers and failed to clear LCMV. Despite a more pronounced defect in degranulation – but not in cytotoxicity – *beige* CTL cleared the virus, while *pearl* CTL had an intermediate effect on virus clearance. Taken together, when CTL from the different HLH-prone mice failed to eliminate LCMV in this short-term protection assay, the mice developed HLH independent from their residual cytolytic activity as determined in a ^51^Cr-release assay *in vitro* (Figure 3D). Thus, this *in vivo* virus protection assay is a better correlate for HLH susceptibility than cytolytic activity of CTL measured *in vitro*, since it translates a gradient of impaired cytotoxicity into the clinically observed “yes-no” decision for the development of HLH.

### HLH disease severity in patients with FHL syndromes

To assess how these observations in the murine cytotoxicity mutants relate to human patients, we intended to determine HLH disease severity in cohorts of patients carrying the respective mutations. Disease severity in humans is, however, not only determined by the affected gene, but also by the nature of the mutation (complete vs. partial loss-of function), genomic heterogeneity, and environmental factors including infections. Since disease severity in human cytotoxicity mutants correlates with the age at onset of HLH (54, 55), we used this as a surrogate parameter. To control in part for the nature of the mutation, we selected only patients with predicted severe impairment of protein expression due to a null mutation, a large gene deletion, the introduction of a stop codon or a frame shift mutations leading to a stop codon in the corresponding genes. We recently published data on patients with severe bi-allelic mutations in the *PERFORIN*, the *SYNTAXIN-11*, and the *RAB27A* genes (44). For this study we added observations in a cohort of patients with mutations in the *LYST* gene that was identified from the literature. Since only one HPS2 patient with an *AP3b1* mutation has been reported to have developed the full picture of HLH (at 5 years of age) (19), we did not include this group of HPS2 patients in our analysis.

**Figure 5:**
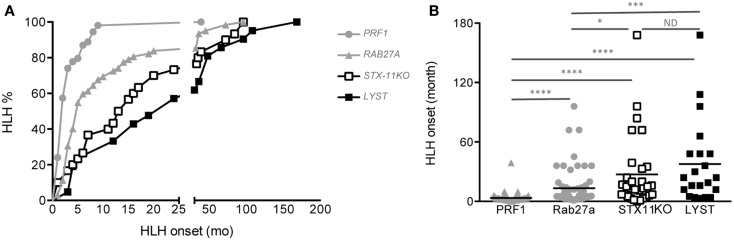
**Delayed HLH onset in patients with *SYNTAXIN-11* and *LYST* deficiency compared with *RAB27A*- and *PRF1*-deficient patients**. **(A)** Cumulative incidence and **(B)** age at onset of HLH in FHL2 (PRF1 deficiency, gray circles; *n* = 72), GS2 (RAB27A deficiency, gray triangles; *n* = 61), FHL4 (STX11 deficiency, open squares; *n* = 30), and CHS (LYST deficiency, black squares; *n* = 21) patients carrying null bi-allelic mutations, as detailed in Table 3. The incidence of HLH was analyzed with a log-rank test; *p < 0.05 for FHL4 vs. GS2 patients; ***p < 0.001 for GS2 vs. CHS patients; ****p < 0.0001 for FHL2 vs. GS2, FHL2 vs. FHL4 and FHL2 vs. CHS; ND = no statistical differences were observed between FHL4 vs. CHS patients. The onset of HLH was analyzed with a one-way ANOVA. *p < 0.05; ***p < 0.001; ***p < 0.0001. Mutations in *PRF1, RAB27A*, and *STX11* are detailed in Ref. (44), and mutations in CHS1/*LYST* are detailed in Table 3.

As expected from our previous study, although there was a high variability in the age at onset for all four diseases, significant differences could be demonstrated between the different cohorts (Figures 5A,B). The mean age of HLH onset was 3.4 ± 5 months in *PERFORIN*-deficient patients, 13.4 ± 19 months in *RAB27A*-deficient patients, 27.3 ± 37 months in *SYNTAXIN-11*-deficient patients and 37.7 ± 41.9 months in *LYST*-deficient patients. Thus, this analysis reveals a gradient of HLH severity in humans that is identical to the mouse models: HPS2 < CHS < FHL4 < GS2 < FHL2.

## Discussion

This study provides a comprehensive comparative analysis of HLH disease severity in mice and humans with mutations in different genes involved in cellular cytotoxicity. While the study builds on a number of previously published observations comparing individual strains of mice or patient cohorts, it is the first study to directly compare a large number of mutant mouse strains, in which HLH is induced and assessed with a single experimental protocol. Notably, we related the clinical HLH symptoms not only to the affected gene, but also to immunobiological parameters such as the degree of the degranulation/cytotoxicity defect of T cells and the ability to provide control of the triggering viral infection.

**Figure 6:**
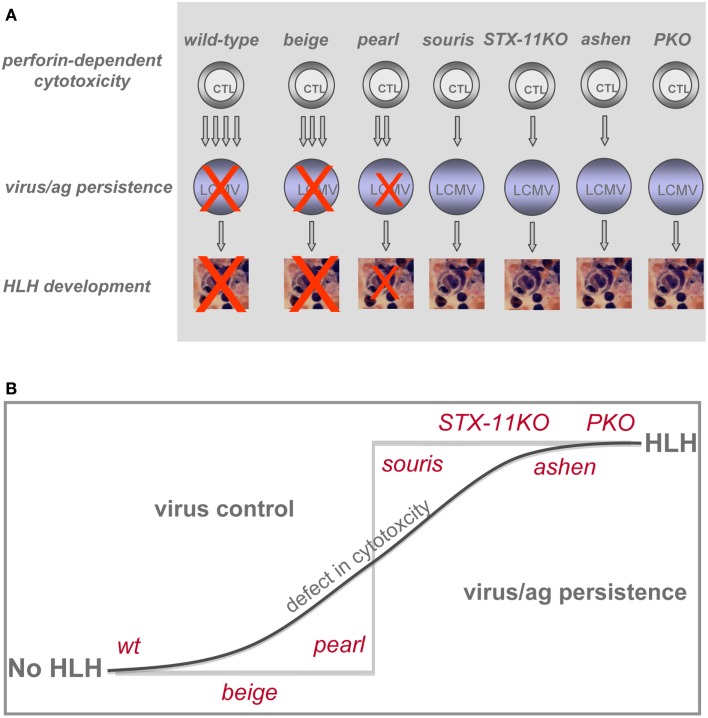
**Impact of various genetic defects on threshold of HLH development**. **(A)** Differences in CTL cytotoxicity and their impact on LCMV control determine whether or not HLH develops. **(B)** Pronounced impairment of CTL cytotoxicity results in loss of virus control and development of HLH.

The overall result is a clear hierarchy of HLH severity among the different genetic defects predisposing to HLH that is surprisingly consistent between humans and mice. This is an important validation of using LCMV infection of murine cytotoxicity mutants for the study of human genetic defects in cytotoxicity and FHL development. While *beige* mice did not develop HLH upon LCMV infection, *pearl* mice showed transient HLH and *souris, STX-11KO, ashen*, and *PKO* mice exhibited the full picture of the disease. Moreover, among the latter four strains a gradient of disease severity could be established when considering the individual HLH associated parameters (Table 2). *Souris* mice had the least weight loss and drop in temperature, while *PKO* mice had the most severe and frequently lethal HLH progression (Figure 1). Finally, we could confirm our previous observation that the disease course was more severe in *ashen* (GS2) than in *STX-11KO* (FHL4) mice. Thus, the gradient of HLH severity in mice was: *wild-type* < HPS2 < CHS < FHL4 < GS2 < FHL2 (Figure 6). Interestingly, the parameters best reflecting this gradient were ferritin and sCD25, while IFN-γ serum levels did not correlate as good, at least when analyzed at day 12 post infection.

In humans, disease severity as assessed by age at onset of HLH followed exactly the same pattern. However, in all cohorts, there were patients who developed HLH in the first months of life, confirming that the affected gene cannot predict disease onset in the individual patient. The different ages at onset probably rather reflect the likelihood of loosing control of perforin-mediated immune homeostasis in response to triggers of different intensity that are encountered by all children.

As expected, none of the mouse strains developed disease spontaneously. Infection with LCMV was required to trigger disease, while other infections including respiratory syncytical virus (RSV) and pneumonia virus of mice (PVM) failed to induce disease, even in the most severe mutant lacking perforin (unpublished observations). Of note, mouse cytomegalovirus (MCMV) is able to induce HLH in *PKO* (56, 57), but not in jinx mice (42). No data on HLH are so far available for the other HLH-prone mouse strains, although a higher susceptibility of *beige* mice to MCMV infection has been reported (58). The potency of LCMV to induce HLH might in part be explained by the fact that it infects antigen-presenting cells such as dendritic cells (63), leading to direct stimulation of the T cell response without the need for cross-presentation. This contributes to the fact that it is a better stimulator of CD8 T cell responses in the mouse than any of the other infections. Of note, this is a property that is also shared by EBV infection in humans, where infected B cells can also serve as APC and will lead to prolonged antigenic stimulation, if they are not eliminated by NK or T cells (64). Overall, a very potent CTL stimulation is required to provoke the impaired immune homeostasis characteristic of HLH in mice. This is in apparent contrast to early-onset HLH in patients with cytotoxicity defects, where in the majority of cases no pathogen trigger can be identified. This could reflect variable and incomplete infectious disease work-up of the patients, which may not only relate to known infections, but could also point to a role for so far unknown novel viruses. Alternatively, it may indicate that in humans, different from mice, perforin-mediated cytotoxicity also plays a role in T cell homeostasis under resting conditions, as it is described for the Fas/FasL pathway (65, 66). Thus, T cell–T cell interactions or T/NK cell–APC interactions involving perforin could be relevant for maintaining immune homeostasis even in the absence of infections or other obvious immune stimulations (67–73). Further research in HLH patients will be needed to address this important issue, since treatment of a triggering infection can be an important component of successful therapy for HLH (37, 74, 75).

What then is the role of the virus in the murine disease models? Our data and previous experiments suggest that it is not just a trigger of a pathogenetic sequence that – once initiated – becomes self-perpetuating and independent of the virus. This is probably best illustrated by the phenotype of *pearl* mice. Within the first 8 days after infection, these mice could not control virus replication and developed HLH symptoms. However, virus elimination was eventually achieved and this led to full recovery from HLH symptoms. Complementary to these findings, chronic antigen application (gp33 peptide injections in 12 h intervals) induced HLH-like symptoms in LCMV-infected *wild-type* mice indicating the critical role of antigen persistence and prolonged antigen presentation for disease development (41). Thus, persistence of cells presenting viral antigen (usually in the context of persisting virus) is the decisive factor for HLH induction in all animal models studied so far. A previous study has suggested that perforin may have an immunomodulatory function that is independent from its role in the clearance of virus and killing of APCs presenting viral antigen, but related to a role of perforin in modulating antigen presentation by DC (76). In this view, HLH development is not dependent on virus persistence *per se* but determined by an enhanced antigen presentation in the context of perforin deficiency. Further experiments are needed to decide whether it is possible to functionally separate antigen persistence from enhanced antigen presentation in this context.

While virus/antigen persistence was associated with perpetuated disease in all HLH-prone mouse strains analyzed, various factors may determine the hierarchy of disease severity in mice with different genetic defects in cellular cytotoxicity. First, early virus control based on differences in residual cytotoxic activity of T and NK cells may be an important factor in determining the severity of initial disease manifestation. The graded virus load in the spleen at day 8 after infection paralleled very well HLH severity in *beige, pearl, souris*, and *STX-11KO* mice. However, differences in splenic virus titers on day 8 could not explain the different HLH severity in *STX-11KO, ashen*, and *PKO* mice. Thus, a so far unsolved question is why differences in HLH severity are observed in these mice, although all of them exhibited virus persistence, which should lead to the same extent of chronic T cell stimulation by presented viral antigens. Early virus spread to other, non-lymphoid organs could be an important factor determining HLH severity. Differences in the spread of virus to key organs like the liver or the brain with subsequent recruitment of highly activated CTL may explain the fact that *PKO* mice die, while the other mutant mouse strains survive (77). Analysis of the early kinetics of virus spread to other organs may help to resolve this issue. Second, differences in the residual cytotoxic activity may not only affect early virus control and spread, but additionally influence effector cell homeostasis via a more or less efficient killing of distinct APC populations. The elimination of certain APC populations may critically determine the activation status and survival of the hyperreactive T cells and act as a rheostat to limit T cell responses (73, 78–81). As recently demonstrated, the elimination of a rare, antigen-presenting DC population by CD8 T cells in a negative feedback loop critically determines the magnitude of the T cell response in a perforin-dependent way after LCMV infection (82). Third, the proteins affected in the various cytotoxicity mutants are involved in different steps of lysosomal trafficking [as discussed for syntaxin-11 and Rab27a (26, 44)], which could also contribute to the quality of antigen presentation and hence indirectly determine T cell activity and HLH progression. This may also be the case for AP3b1 known to regulate several processes involved in antigen recognition/processing/presentation, i.e., CD1b presentation of phagocytosed antigens and TLR recruitment to phagosome (83, 84). Along the same line, defects in proteins involved in perforin-mediated cytotoxicity may have additional functions in other immune cell types like platelets, neutrophils, and mast cells, which can be relevant for LCMV specific immune responses and thus modulate HLH pathogenesis (33, 85, 86). Fourth, different susceptibility to T cell exhaustion in the various cytotoxicity mutants can modify HLH progression and determine survival. As recently demonstrated, effector T cells chronically exposed to antigenic stimulation showed a variable extent of exhaustive differentiation in different HLH-prone mouse strains. Initially, *STX-11KO* mice developed all diagnostic symptoms of HLH after LCMV infection comparable to *PKO* mice. However, in *STX-11KO* mice with more extensive T cell exhaustion the HLH disease course was attenuated and the mice survived, whereas *PKO* mice developed lethal HLH (43). T cell exhaustion in *STX-11KO* mice was characterized by sustained expression of inhibitory receptors, step-wise loss of effector functions, and finally deletion of the disease-mediating T cells. Thus, T cell exhaustion can be an important disease-modifying parameter in HLH.

In summary, following a defined viral stimulus, the degree of impairment of CTL cytotoxicity was the best predictor of HLH development in the described animal models, but other host factors contributed. HLH appeared to be a threshold disease. Up to a certain degree of impaired cytotoxicity, disease was mild and transient, but resolved once the delayed virus control had been achieved. However, subtle differences in CTL cytotoxicity allowing the establishment of viral persistence led to the full picture of HLH and persistent disease (Figure 6A). Thus, the graded differences in cytotoxicity translated into a “yes-no” phenotype (Figure 6B) with respect to HLH. In HLH patients it is still a matter of debate to which extent CTL or NK cells contribute to disease induction. Hence, this concept may apply in a more general form to cytotoxic lymphocytes. Depending on the initial trigger CTL and/or NK cells may play the critical role in HLH induction and progression. While restoration of cytotoxicity and virus control appear to be key variables in the causal treatment of the disease, a further investigation of these factors in humans and mice may point to additional treatment approaches for this highly aggressive syndrome.

## Materials and Methods

### Patients

All patients with FHL or GS2 diagnosis were previously reported [summarized in Ref. (44)]. Patients with CHS diagnosis were previously published (as referenced in Table 3).

**Table 3:** **Genotype and age at HLH onset of previously published patients carrying severe bi-allelic mutations in *CHS1/LYST***.

LYST mutations	Predicted effect	*n*	HLH onset (months)	Reference
c.2620delT	p.F874Ffs25X	1	66	Certain et al. (59)
c.C3310T	p.R1104X	1	30	Certain et al. (59)
c.7555delT	p.Y2519Ifs9X	1	168	Certain et al. (59)
c.del7060-7066	p.delL2354_D2356Mfs15X	1	19	Certain et al. (59)
c.5317delA*/c.9228 + 10bp ins*	p.R1773Dfs12X*/p.H3076Hfs8X*	1	16	Certain et al. (59)
c.del9106-9161	p.delG3036_S3054Gfs15X	1	24	Certain et al. (59)
c.9590delA	p.Y3197Lfs61X	1	12	Certain et al. (59)
c.5004delA	p.G1668Gfs28X	1	12	Scherber et al. (60)
c.5519delC	p.S1840Yfs1X	1	108	Scherber et al. (60)
c.9590delA	p.Y3197Lfs61X	1	3	Scherber et al. (60)
c.3622C > T*/c.11002G > T*	p.Q1208X*/E3668X*	1	16	Scherber et al. (60)
c.5506C > T	p.R1836X	1	48	Kaya et al. (61)
c.5506C > T	p.R1836X	1	4	Kaya et al. (61)
IVS24 c.7060-1G > A	Exon25fsX	1	4	Jessen et al. (46)
c.10551_10552del2	p.Y3517X	1	5	Jessen et al. (46)
c.5506C > T	p.R1836X	1	4	Jessen et al. (46)
c.2374_2375 delGA	p.D792FX6	1	96	Jessen et al. (46)
c.4508C > G	p.S1483X	1	48	Jessen et al. (46)
c.4508C > G	p.S1483X	1	36	Jessen et al. (46)
c.5506C > T	p.R1836X	1	48	Jessen et al. (46)
10395delA	p.K3465Kfs2X	1	24	Karim et al. (62)

*All of the selected genetic anomalies were either large deletion, null mutations, introduce a stop codon or affect the first base of an intron predicted to induce a frameshift, and a consecutive stop codon*.

*Heterozygous mutations; *n* = number of patients.

### Mice and virus

C57BL/6 (*wild-type*, wt) mice were purchased from Charles River Laboratories (Sulzfeld, Germany). C57BL/6J-*Lystbg^J^*/J (*beige^J^*; stock no. 000629) and B6Pin.C3-*Ap3b1^pe^*/J (*pearl*, stock no. 003215) mice were purchased from the Jackson Laboratory (Bar Harbor, USA), and C57BL/6-*Lyst^bg-Btlr^*/Mmcd (*souris*; stock no. 010470-UCD) mice originally generated by Dr. B. Beutler and colleagues (Scripps Research Institute, La Jolla, CA, USA) were obtained from the Mutant Mouse Regional Resource Center (University of California, Davis, CA, USA). Syntaxin-11-deficient (*STX-11KO)* mice were generated by Dr. U. zur Stadt (Hamburg) on a C57BL/6 background by deletion of the only coding exon. C3H/HeSn-Rab27a^ash^/J mice were purchased from the Jackson Laboratory and backcrossed to the C57BL/6 background for 10 generations (C57BL/6J-Rab27a^ash^/j; *ashen*). Perforin-deficient C57BL/6-Prf1^tm1Sdz^ (*PKO*) mice were obtained from Dr. H. Hengartner (Zurich). Mice were kept under specific pathogen-free conditions. All mouse experiments were approved by the Regierungspraesidium Freiburg. The lymphocytic choriomeningitis virus WE (LCMV-WE) was grown on MC57G fibroblasts and stored at −80°C until use. Mice were injected intravenously with 200 pfu (*plaque forming units*). To quantify virus in organs from infected mice a focus forming assay was used as described (87). Temperatures were obtained using a digital infrared ear thermometer (Braun, ThermoScan type 6022).

### HLH biomarkers in mice

Blood counts were determined by a Sysmex KX-21 hematology analyzer. Serum levels of ferritin, triglycerides, LDH, and GLDH were analyzed by the Department of Clinical Chemistry using the Roche Modular Analytics Evo. Levels of sCD25 were determined by using the mouse IL-2Ralpha DuoSet kit (R&D systems) according to the instructions of the manufacturer. The IFN-γ ELISA was performed as described before (46).

### Histology

To evaluate hemophagocytic macrophages, immunohistochemistry on paraffin-embedded liver sections was performed as previously described (46).

### Antibodies, intracellular staining, degranulation, and cytotoxicity assay

Antibodies were purchased from eBioscience or BD Biosciences. Surface expression of CD107a and intracellular IFN-γ of CD8^+^CD3^+^ CTL was determined after 4 h of restimulation with the immunodominant CTL epitope gp33-41 (PolyPeptide) or medium in the presence of monensin (BD Biosciences). For fixation and permeabilization of spleen cells the Cytofix/Cytoperm kit (BD Biosciences) was used. CTL cytotoxicity was determined in a 5 h ^51^chromium-release assay by incubating spleen cells as effectors with LCMV-infected MC57 target cells. In order to calculate the CTL to target ratio, CD8 T cells were quantified by antibody staining and flow cytometry.

### Adoptive transfer experiment

Splenic CD8 T cells from mice that had been infected with 200 pfu LCMV-WE 8 days earlier were MACS purified using the MACS CD8a^+^ T Cell Isolation Kit II (Miltenyi Biotec). Purity was determined by flow cytometry and was above 90% in all experiments. About 2 × 10^6^ purified CD8 T cells were transferred intravenously into C57BL/6 *wild-type* mice that had been infected with 10^4^ pfu LCMV 10 h before. After 18 h of adoptive cell transfer, splenic virus titers were determined.

### Statistical analysis

Tests were performed using the GaphPad InStat software version 3.06. The comparison between data was evaluated with a one-way ANOVA (Analysis Of Variance) with posttest. Differences were considered significant at a *p*-value below 0.05.

## Authors Contribution

Birthe Jessen, Tamara Kögl, and Fernando E. Sepulveda performed experiments; Stephan Ehl, Peter Aichele, Genevieve de Saint Basile designed the study and supervised the project; Stephan Ehl, Peter Aichele, Genevieve de Saint Basile, and Birthe Jessen wrote the manuscript.

## Conflict of Interest Statement

The authors declare that the research was conducted in the absence of any commercial or financial relationships that could be construed as a potential conflict of interest.

## Appendix

**Table A1:** **Analysis of HLH biomarker on day 8 after LCMV infection**.

HLH parameter	Wild-type	Beige	Pearl	Souris	Syntaxin-11KO	Ashen	PKO
Ferritin (ng/mL)	393.8 ± 244.0	493.9 ± 181.1	**892.6 ± 424.6*****	481.8 ± 259.4	365.0 ± 163.1	578.3 ± 79.7	**1280.7 ± 1212.9*****
sCD25 (pg/mL)	545.7 ± 206.2	508.0 ± 261.5	805.2 ± 294.5	816.4 ± 234.5	707.1 ± 389.7	916.7 ± 415.3	**1060.4 ± 314.1***
Triglycerides (mg/dL)	58.3 ± 18.0	97.1 ± 21.6	97.3 ± 93.3	63.5 ± 54.2	**187.5 ± 97.0***	174.0 ± 107.9	**164.1 ± 90.1*****
Additional parameter
IFN-γ (ng/mL)	0.4 ± 1.2	8.45 ± 9.9	**20.5 ± 16.5***	**114.8 ± 26.0*****	**79.7 ± 60.6****	**70.3 ± 42.3***	**156.7 ± 56.8*****
**HLH**	**NO**	**NO**.	**Transient**	**YES**	**YES**	**YES**	**Lethal**

*Gray fields indicate fulfilled HLH criteria*.

*The red font accentuates the final disease phenotype*.

*****p* < 0.001; ***p* < 0.01; **p* < 0.05 (ANOVA; statistically significant differences compared to day 8 values of wild-type mice)*.
